# Precision patient selection for ablate-and-pace: a multi-center study comparing leadless pacemaker vs. standard pacemaker in patients with indication to ablate-and-pace for atrial fibrillation (LEAD-AP)

**DOI:** 10.3389/fcvm.2026.1741074

**Published:** 2026-02-16

**Authors:** Ingrid Overeinder, Luigi Pannone, Domenico Laviola, Wael Zaher, Giacomo Mugnai, Gianmarco Arabia, Ivan Eltsov, Ioannis Doundoulakis, Domenico Giovanni Della Rocca, Paul-Adrian Calburean, Vedran Pašara, Ludovica Carmagnola, Tommaso Sattin, Antonio Sorgente, Alvise Del Monte, Giacomo Talevi, Gezim Bala, Alexandre Almorad, Erwin Ströker, Juan Sieira, Ali Gharaviri, Mark La Meir, Pedro Brugada, Andrea Sarkozy, Gian Battista Chierchia, Antonio Curnis, Serge Boveda, Carlo de Asmundis

**Affiliations:** 1 Heart Rhythm Management Centre, Universitair Ziekenhuis Brussel, Heart Rhythm Research Brussels, Postgraduate Program in Cardiac Electrophysiology and Pacing, Vrije Universiteit Brussel, European Reference Networks Guard-Heart, Brussels, Belgium; 2 Heart Rhythm Management Department, Clinique Pasteur, Toulouse, France; 3 Division of Cardiology, Cardio-Thoracic Department, University Hospital of Verona, Verona, Italy; 4 Institute of Cardiology, ASST Spedali Civili, Department of Medical and Surgical Specialties, Radiological Sciences and Public Health, University of Brescia, Brescia, Italy; 5 Cardiac Surgery Department, Universitair Ziekenhuis Brussel—Vrije Universiteit Brussel, Brussels, Belgium

**Keywords:** ablate-and-pace, atrial fibrillation, atrioventricular node ablation, leadless pacemaker, transvenous pacemaker

## Abstract

**Background:**

In patients with symptomatic permanent atrial fibrillation (AF) who are not candidates for rhythm control, atrioventricular node (AVN) ablation followed by ventricular pacing “ablate-and-pace” offers reliable rate control and symptom relief. Conventional transvenous systems are effective but associated with lead- and pocket-related complications. Leadless pacemakers represent a promising alternative, yet comparative data in this setting remain scarce. The aim is to compare clinical outcomes of leadless vs. transvenous single-chamber pacemakers in patients undergoing AVN ablation for permanent AF.

**Methods:**

We conducted a retrospective, multicenter study (LEAD-AP) of 168 consecutive patients undergoing ablate-and-pace between 2,017 and 2024 across four European centers. Patients received either a leadless pacemaker (*n* = 56) or a conventional transvenous VVI pacemaker (*n* = 112). The primary efficacy endpoint was the composite of all-cause mortality, cardiovascular mortality, AF-related hospitalizations, unplanned visits and device-related hospitalizations or reinterventions. The secondary efficacy endpoint was device-related hospitalizations or reinterventions. The primary safety endpoint was acute complications within 30 days.

**Results:**

Patients in the leadless group more frequently underwent a single-step ablate-and-pace strategy (96.4% vs. 10.9%, *p* < 0.001), resulting in shorter hospitalization (1.1 days ± 3.1 vs. 5.7 days ± 2.2, *p* = 0.008). At 24 months of follow-up, there was no statistically significant difference between patients with leadless pacemaker vs. standard single-chamber VVI pacemaker in the event-free survival for the clinical efficacy endpoint (82.1% vs. 80.4% Log-Rank *p* = 0.29).

**Conclusions:**

Leadless pacemakers provide comparable safety and efficacy to transvenous systems in ablate-and-pace patients, while enabling shorter hospitalization through a streamlined single-step approach.

## Graphical Abstract

**Figure:**
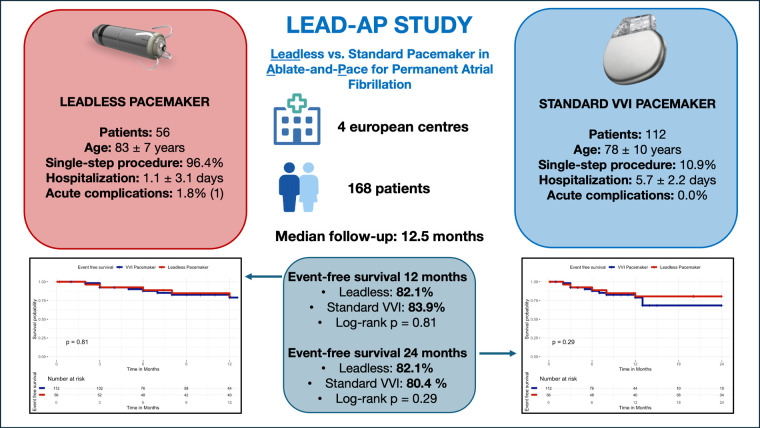

## Introduction

Atrial fibrillation (AF) is the most common sustained cardiac arrhythmia in adults, affecting approximately 2%–4% of the general population, and is associated with a significantly increased risk of heart failure, stroke, and all-cause mortality (1, 2). In patients with symptomatic permanent AF, a rate control strategy is preferred when rhythm control is no longer pursued or effective (3). Among the rate control options, atrioventricular node (AVN) ablation combined with pacemaker implantation, commonly referred to as “ablate-and-pace” strategy, is effective in achieving stable ventricular rate control and symptoms relief (1). This strategy can be performed either as a single-step procedure, combining AVN ablation and pacemaker implantation in the same session, or as a staged approach, with the two interventions performed on separate days. While the staged approach may offer procedural flexibility, the single-step method allows for streamlined management and reduced hospitalization time, particularly when using leadless systems via femoral access (4).

Traditionally, the ablate-and-pace involves the implantation of a transvenous right ventricular pacemaker (5). However, this approach is associated with a complication rate as high as 9.5% including: lead-related complications, infections, hematomas, pneumothorax, pericardial effusion and issues related to device longevity (4). The recent advent of leadless pacemaker systems, such as the Micra™ Transcatheter Pacing System, has offered a less invasive alternative, potentially reducing procedure-related morbidity. Although several observational studies have explored the safety and efficacy of leadless pacing (6–8) data comparing leadless vs. standard transvenous pacing systems for ablate-and-pace indication remain scarce (9). The LEAD-AP study was therefore designed as a multi-center study to compare the outcomes of leadless pacemaker implantation vs. standard single-chamber transvenous pacemaker implantation in patients undergoing AVN ablation for symptomatic permanent AF. Beyond a simple device comparison, this study proposes a structured clinical phenotyping framework for device and strategy selection in the ablate-and-pace setting, representing a foundational step toward operational precision cardiology at the bedside. While the present analysis is based on conventional clinical variables, such a framework is inherently compatible with future integration of genomic markers, multi-omics risk signatures, and machine learning–derived prediction models that could further refine individualized device choice and procedural strategy.

## Methods

### Study design and population

LEAD-AP is a retrospective, multi-center clinical study conducted at: 1) Heart Rhythm Management Centre of Universitair Ziekenhuis Brussel (UZ Brussel), Belgium, 2) Heart Rhythm Management Department, Clinique Pasteur, Toulouse, France, 3) Department of Cardiology, University Hospital of Verona, Verona, Italy and 4) Institute of Cardiology, ASST Spedali Civili, Department of Medical and Surgical specialties, Radiological sciences and Public Health, University of Brescia, Brescia, Italy.

All consecutive patients undergoing ablate-and-pace by either a leadless pacemaker or a conventional single-chamber transvenous pacemaker between February 2017 and July 2024, were retrospectively screened and enrolled if they met the following inclusion criteria: 1) diagnosis of symptomatic permanent AF with indication to ablate-and-pace; 2) ablate-and-pace performed with leadless pacemaker or conventional single-chamber right ventricle apex transvenous pacemaker. Key exclusion criteria included: 1) age <18 years; 2) left bundle branch pacemaker or biventricular CRT pacemaker; 3) transient or reversible causes of AF; 4) planned rhythm control interventions; 5) QRS duration ≥130 ms; 6) left ventricular ejection fraction on the most recent pre-procedural echocardiogram <50%; 7) presence of intracardiac masses; 8) contraindications to anticoagulation or pacemaker therapy; 9) active malignancy requiring treatment; 10) life expectancy <6 months and inability or unwillingness to comply with follow-up. The clinical data were collected on electronic medical records. Data were carefully reviewed and confirmed by 2 independent researchers (L.P. and D.L.), both blinded to cardiac arrhythmia recurrence, to guarantee the accuracy of the data extraction. The protocol adheres to the Declaration of Helsinki as revised in 2013 and was approved by the local institutional ethics committee. Written informed consent was obtained from all participants prior to enrollment.

### Ablation and device implantation procedure

AVN ablation and pacemaker implantation were performed as a single or staged procedure, at the discretion of the treating physician. In all patients, anticoagulation was managed according to current guidelines, and continued uninterrupted during the procedure whenever possible. Devices in both arms were programmed to VVI mode at a lower rate of 70 bpm.

Patients received either a leadless pacemaker (Micra™ VR, Medtronic) or a standard single-chamber transvenous pacemaker (Medtronic Azure XT SR MRI with CapSure Fix Novus MRI lead), both in combination with AVN ablation.

**Figure 1:**
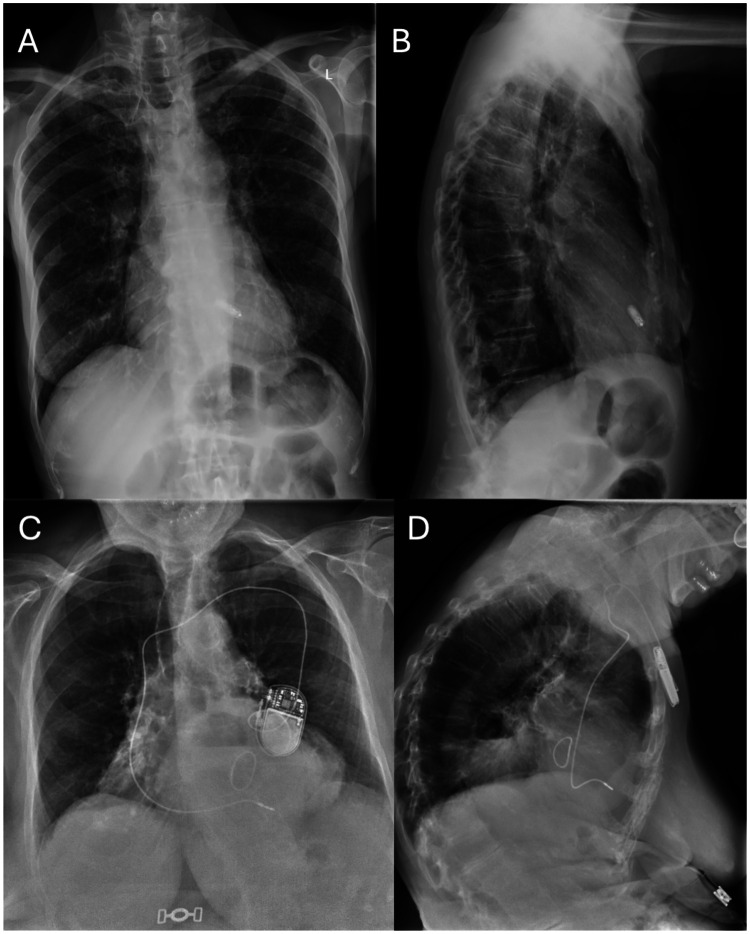
Chest x-Ray of patients with leadless pacemaker vs conventional single-chamber transvenous pacemaker. Chest x-Ray of patients with leadless pacemaker vs conventional single-chamber transvenous pacemaker. **(A)** Chest x-Ray of a patient with leadless pacemaker (postero-anterior view); **(B)** Chest x-Ray of a patient with leadless pacemaker (latero-lateral view); **(C)** Chest x-Ray of a patient with a conventional single-chamber transvenous pacemaker (postero-anterior view); **(D)** Chest x-Ray of a patient with a conventional single-chamber transvenous pacemaker (latero-lateral view).

In the leadless group, the Micra™ VR system was implanted via femoral access following manufacturer instructions. In the standard pacemaker group, a transvenous single-chamber device was implanted via subclavian or axillary access. In both groups, a remote home monitoring system was activated to collect device data and facilitate early detection of complications, Figure 1.

### Study objectives and endpoints

The primary efficacy endpoint was the composite incidence of all-cause mortality, cardiovascular mortality, AF-related hospitalizations, unplanned outpatient visits for AF, and device-related hospitalizations or reinterventions (10). The secondary efficacy endpoint was the incidence of device-related hospitalizations or reinterventions (including loss of pacing function, lead dislodgement, battery change, device infection, hematoma, vascular access complications, and any revision or replacement procedure). The primary safety endpoint was the occurrence of acute complications within 30 days post-implantation, including death, pericardial effusion, cardiac tamponade, pneumothorax, device infection, and vascular access complications.

### Follow-up

Patients were followed with outpatient visits scheduled at 1, 3, 6, 12, 18, and 24 months post-implantation. Follow-up assessments included clinical examination, 12-lead ECG, device interrogation, and systematic documentation of adverse events, reinterventions, and hospitalizations.

### Statistical analysis

Continuous variables were assessed for normality using the Shapiro–Wilk test. Normally distributed variables were reported as mean ± standard deviation and compared using unpaired or paired t-tests, as appropriate. Non-normally distributed variables were expressed as median (interquartile range) and compared using Mann–Whitney *U* or Wilcoxon tests. Categorical variables were summarized as frequencies (percentages) and compared using the chi-squared test or Fisher's exact test.

Kaplan–Meier survival curves were constructed to evaluate time-to-event outcomes, and comparisons between groups were made using the log-rank test. Cox proportional hazards regression was performed to assess hazard ratios (HRs) with 95% confidence intervals (CIs) for primary and secondary outcomes. All statistical analyses were performed using R statistical software (R Foundation for Statistical Computing, Vienna, Austria). A *p*-value < 0.05 was considered statistically significant.

## Results

### Study population characteristics

A total of 168 patients undergoing ablate-and-pace for symptomatic permanent AF were included in the analysis. Among them, 56 patients (33.3%) received a leadless pacemaker and 112 patients (66.7%) received a standard single-chamber transvenous pacemaker. Baseline patient characteristics according to device type are summarized in Table 1.

**Table 1:** Patient characteristics.

Variables	Transvenous pacemaker (*N* = 112)	Leadless pacemaker (*N* = 56)	Total (*N* = 168)	*p* value
Age (Years)	77.6 (9.8)	83.2 (7.4)	79.5 (9.5)	<0.001
Gender (male)	52 (46.4%)	24 (42.9%)	76 (45.2%)	0.74
Hypertension (*N*,%)	68 (60.7%)	50 (89.3%)	118 (70.2%)	<0.001
Diabetes (*N*,%)	20 (17.9%)	14 (25.0%)	34 (20.2%)	0.31
Dylipidemia (*N*,%)	38 (33.9%)	16 (28.6%)	54 (32.1%)	0.59
Coronary artery disease (*N*,%)	32 (28.6%)	18 (32.1%)	50 (29.8%)	0.72
Peripheral vascular disease (*N*,%)	10 (8.9%)	2 (3.6%)	12 (7.1%)	0.34
Major bleeding (*N*,%)	14 (12.5%)	8 (14.3%)	22 (13.1%)	0.81
HFpEF (*N*,%)	32 (28.6%)	20 (35.7%)	52 (31.0%)	0.38
NYHA class (*N*,%)				0.24
1	18 (16.1%)	6 (10.7%)	24 (14.3%)	
2	50 (44.6%)	26 (46.4%)	70 (41.7%)	
3	44 (39.3%)	24 (42.9%)	68 (40.5%)	
4	0 (0.0%)	0 (0.0%)	0 (0.0%)	
Echo LVEF (%)	57.5 (53–62)	60.7 (55–66)	58.5 (54.0–63)	0.36
ECG QRS (ms)	102.4 ± 18.6	98.7 ± 16.3	101.1 ± 17.8	0.21
eGFR (mL/min)	51.6 (19.5)	56.9 (14.3)	52.9 (18.5)	0.14
Creatinine (mg/dl)	1.2 (0.5)	1.2 (0.7)	1.2 (0.6)	0.858
OAC (*N*,%)	112 (100.0%)	56 (100.0%)	168 (100.0%)	1.0
AAD class I (*N*,%)	0 (0.0%)	0 (0.0%)	0 (0.0%)	1.0
Beta blockers (*N*,%)	86 (76.8%)	42 (75.0%)	128 (76.2%)	0.85
AAD class III (*N*,%)	30 (26.8%)	14 (25.0%)	44 (26.2%)	0.85
AAD class IV (*N*,%)	8 (7.1%)	4 (7.1%)	12 (7.1%)	1.0
Ablate-and-pace single step (*N*,%)	10 (10.9%)	54 (96.4%)	64 (43.2%)	< 0.001

AAD, antiarrhythmic drug; HFpEF, heart failure with preserved ejection fraction; OAC, oral anticoagulant; TIA, transient ischemic attack.

Patients in the leadless group were significantly older at the time of the procedure (83.2 ± 7.4 years vs. 77.6 ± 9.8 years, *p* < 0.001) and had a higher prevalence of hypertension (89.3% vs. 60.7%, *p* < 0.001).

There were no other significant differences in baseline characteristics, including: sex, diabetes, dyslipidemia, coronary artery disease, peripheral vascular disease, bleeding or the use of *β*-blockers, class III/IV antiarrhythmic drugs, see Table 1.

### Procedure characteristics

Patients in the leadless group underwent a single-step procedure more frequently compared with the standard pacemaker group (96.4% vs. 10.9%, *p* < 0.001). The total combined duration of hospitalization for the ablate-and-pace (ablatio*n* + implant) strategy was lower in the leadless pacemaker compared to standard pacemaker (1.1 ± 3.1 days vs. 5.7 ± 2.2 days, *p* = 0.008). The total combined duration of hospitalization for the ablate-and-pace (ablatio*n* + implant) strategy was lower in the single step group compared to two steps group (1.3 ± 3.4 days vs. 5.5 ± 2.1 days, *p* = 0.009). The rate of acute complications within 30 days post-implantation (primary safety endpoint) was low and not different between the two groups, 1/56 patients (1.8%) in the leadless group and 0/112 (0%) in the standard group (*p* = 0.33). In the leadless group, the only acute complication was a vascular access site hematoma/bleeding. In the standard group, no acute complications were recorded. No procedure-related deaths occurred in either group.

### Follow-up

After a median follow-up of 12.5 months [IQR: 7.0–39.3], the incidence of the secondary endpoint of device-related hospitalizations or reinterventions was 10.7% in the leadless group (6/56 patients) and 8.9% in the standard group (10/112 patients, Log-Rank *p* = 0.78).

**Figure 2:**
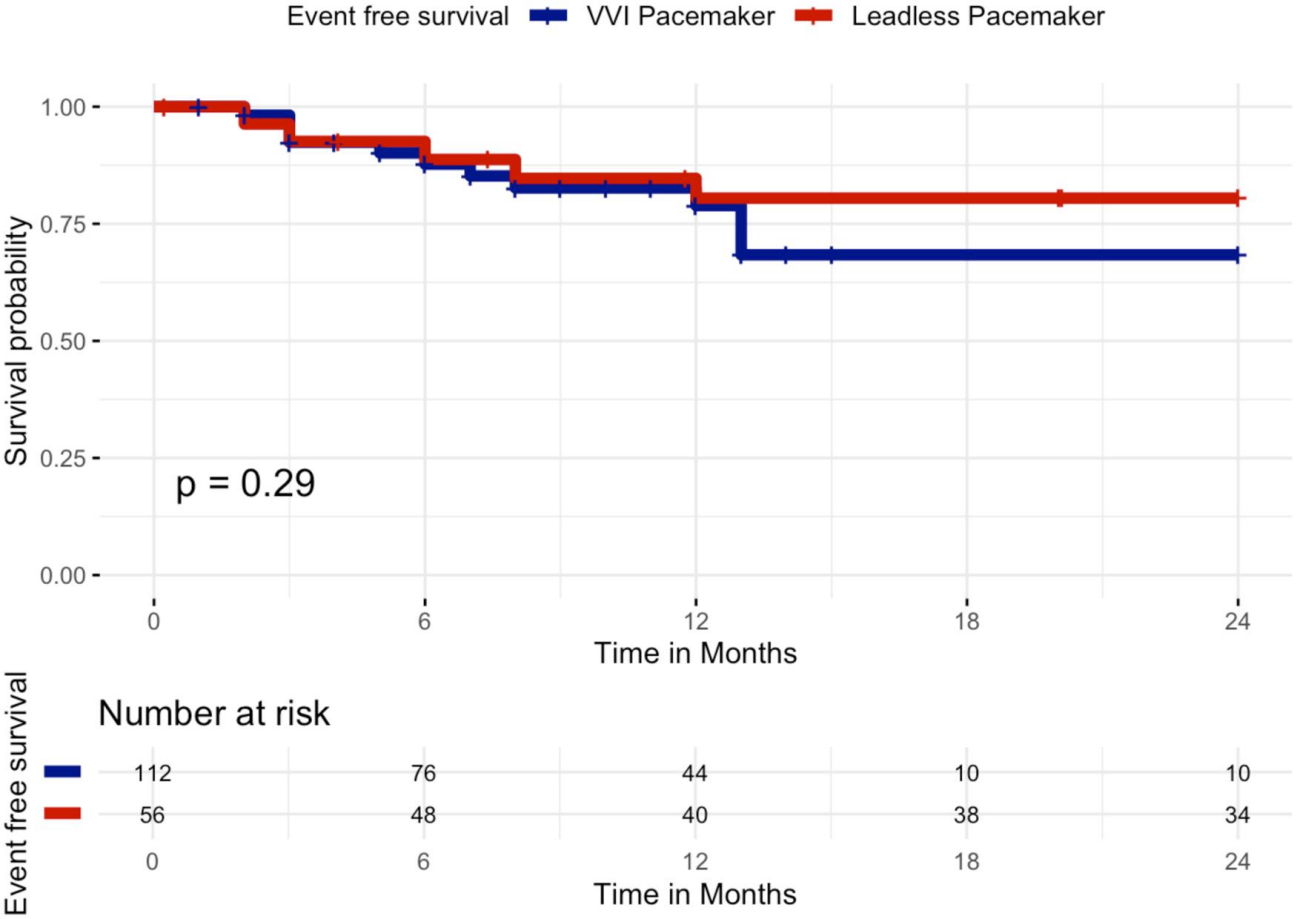
Kaplan–Meier curves of survival free from the primary composite clinical efficacy endpoint by VVI pacemaker or leadless pacemaker at 24 months. Kaplan–Meier curves showing event-free survival for the primary composite clinical efficacy endpoint (all-cause mortality, cardiovascular mortality, atrial fibrillation–related hospitalizations, unplanned AF outpatient visits, or device-related hospitalizations/reinterventions) over 24 months of follow-up in patients implanted with a leadless pacemaker (red curve) vs. a transvenous VVI pacemaker (blue curve). No significant difference was observed between the two groups (log-rank *p* = 0.29).

At 24 months of follow-up, Kaplan–Meier analysis of event-free survival showed no statistically significant difference between patients receiving a standard single-chamber VVI pacemaker and those implanted with a leadless pacemaker. Event-free survival for the primary composite clinical efficacy endpoint (all-cause mortality, cardiovascular mortality, atrial fibrillation–related hospitalizations, unplanned AF outpatient visits, or device-related hospitalizations/reinterventions) was 82.1% in the leadless group (46/56 patients free from events) and 80.4% in the VVI group (90/112 patients free from events), Log-Rank *p* = 0.29, Figure 2. In particular, the primary outcome occurred in 32 patients, 10/56 patients in the leadless group and 22/112 patients in the VVI group: death occurred in 4/56 patients in the leadless group (7.1%) and 10/112 patients in the VVI group (8.9%), all deaths were adjudicated as cardiovascular. Atrial fibrillation-related hospitalization occurred in 0/56 patients in the leadless group and 1/112 patients in the VVI group (0.9%). Unplanned AF outpatient visits occurred in 0/56 patients in the leadless group and 1/112 patients in the VVI group (0.9%). Device-related hospitalizations or reinterventions incidence was 10.7% in the leadless group (6/56 patients) and 8.9% in the standard group (10/112 patients).

**Figure 3:**
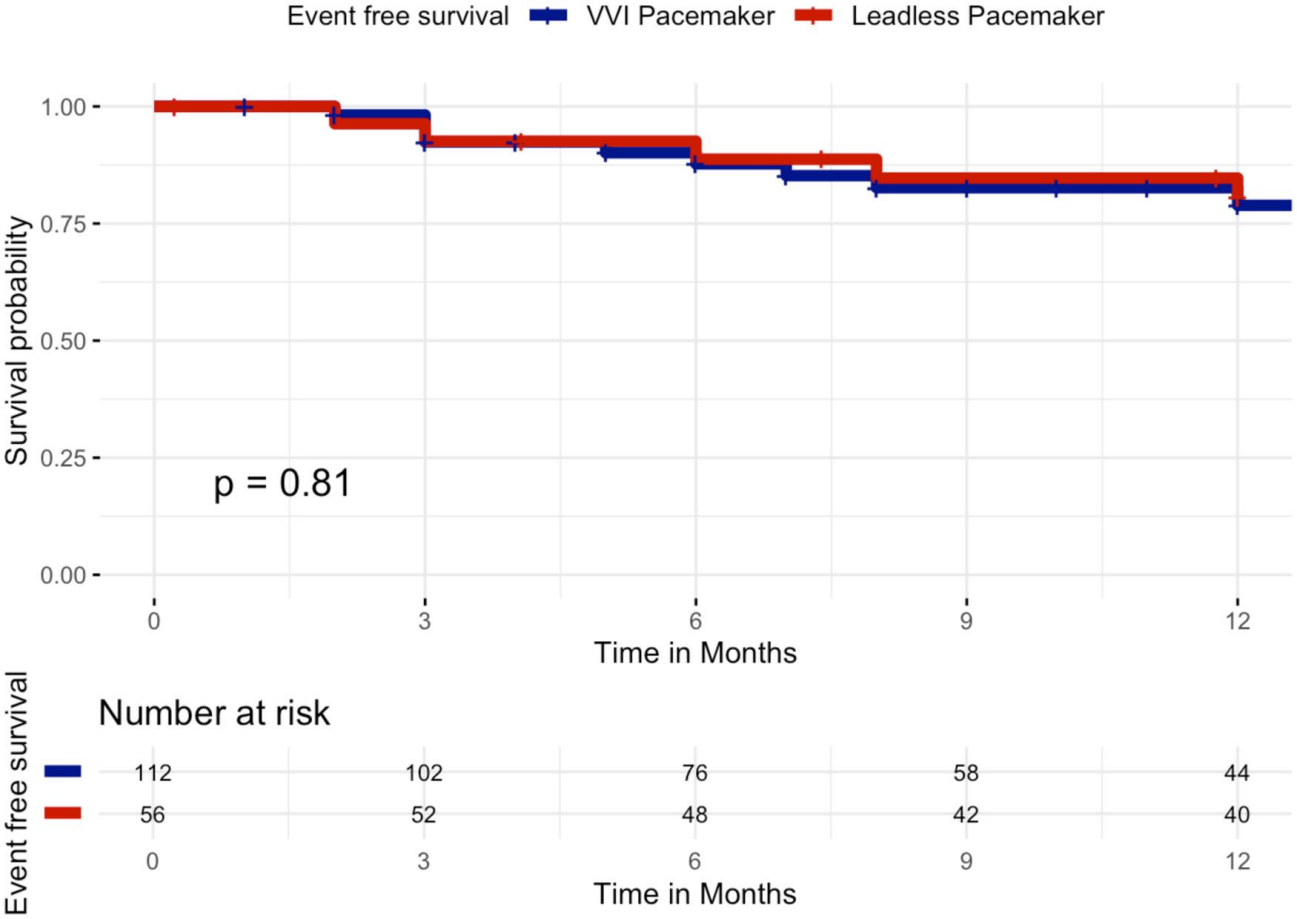
Kaplan–meier curves of survival free from the primary composite clinical efficacy endpoint by VVI pacemaker or leadless pacemaker at 12 months. Kaplan–Meier curves showing event-free survival for the primary composite clinical efficacy endpoint (all-cause mortality, cardiovascular mortality, atrial fibrillation–related hospitalizations, unplanned AF outpatient visits, or device-related hospitalizations/reinterventions) over 12 months of follow-up in patients implanted with a leadless pacemaker (red curve) vs. a transvenous VVI pacemaker (blue curve). No significant difference was observed between the two groups (log-rank *p* = 0.81).

When restricting the analysis to the first 12 months after implantation, results remained consistent, with no significant difference in event-free survival between groups. At 12 months, survival free from the primary composite endpoint was 82.1% in the leadless group (46/56 patients) and 83.9% in the VVI group (94/112 patients), Log-Rank *p* = 0.81, Figure 3.

### Predictors of the primary composite efficacy endpoint

Cox univariate and multivariate analysis is summarized in Table 2. At univariate Cox regression, significant (not independent) predictors of the primary composite clinical efficacy endpoint included: major bleeding [2.88 (1.28–6.49), *p* = 0.011] and AAD class I [3.39 (1.02–11.25), *p* = 0.047]. At multivariate Cox regression analysis, there was no independent predictor of the primary composite clinical efficacy endpoint.

**Table 2:** Cox univariate and multivariate analysis for predictors of the primary composite clinical efficacy endpoint.

Variables	Cox univariate analysis HR (CI 95%), *p* value
Age	0.99 (0.96–1.04), *p* = 0.96
Gender (male)	0.98 (0.44–2.15), *p* = 0.96
Hypertension	0.67 (0.33–1.37), *p* = 0.28
Diabetes	0.86 (0.37–1.98), *p* = 0.73
Dylipidemia	1.50 (0.73–3.08), *p* = 0.27
Coronary artery disease	1.75 (0.82–6.84), *p* = 0.35
Peripheral vascular disease	2.17 (0.76–6.22), *p* = 0.15
Major bleeding	2.88 (1.28–6.49), *p* = 0.011
HFpEF	1.36 (0.68–2.70), *p* = 0.38
Creatinine	0.55 (0.26–1.18), *p* = 0.13
eGFR	0.99 (0.97–1.01), *p* = 0.45
AAD Class I	3.39 (1.02–11.25), *p* = 0.047
Beta Blockers	2.02 (0.78–5.21), *p* = 0.12
AAD Class III	0.74 (0.31–1.79), *p* = 0.50
AAD Class IV	0.69 (0.16–2.88), *p* = 0.60
Ablate-and-pace single step	0.51 (0.23–1.12), *p* = 0.09
Device (leadless pacemaker vs standard single-chamber VVI pacemaker)	0.60 (0.34–1.38), *p* = 0.14

AAD, antiarrhythmic drug; HFpEF, heart failure with preserved ejection fraction; OAC, oral anticoagulant; TIA, transient ischemic attack.

## Discussion

The main findings of this study can be summarized as follows: (1) Ablate-and-pace strategy is safe and effective with both leadless pacemaker and standard transvenous pacemaker; (2) There is no significant difference in acute complications or long-term outcomes between leadless pacemaker and standard transvenous pacemaker in the context of ablate-and-pace; (3) The total combined duration of hospitalization for ablate-and-pace is lower in the leadless pacemaker due to higher use of a single-step procedure.

### Ablate-and-pace strategy

Among rate control strategies for AF, the ablate-and-pace approach is indicated in patients with symptomatic AF and inadequate ventricular rate control despite medical therapy. In patients with narrow QRS and preserved left ventricular ejection fraction (LVEF >50%), right ventricular (RV) pacing is recommended (Class IIa) (11). This strategy ensures stable rate control and regularizes ventricular response, leading to symptom improvement (12). Notably, randomized data demonstrated reductions in mortality and hospitalizations in patients undergoing AVN ablation compared to those managed with pharmacological therapy alone (5). Despite this established benefit, the optimal pacing modality after AVN ablation remains debated. Conventional transvenous systems are widely available and effective but carry an inherent risk of lead- and pocket-related complications, including infection, vascular obstruction, and device malfunction (13). Leadless pacemakers were specifically designed to address these shortcomings, eliminating the subcutaneous pocket and transvenous lead while providing reliable ventricular capture (6, 14). In our multicenter experience, both approaches demonstrated comparable safety and efficacy profiles, underscoring that the prognostic impact in this population may be driven more by patient comorbidities than by device type. Importantly, our findings confirm that leadless pacing can be performed safely in the ablate-and-pace setting, offering electrophysiologists a feasible alternative to conventional single-chamber VVI systems.

#### Comparison of leadless and transvenous pacemakers in ablate-and-pace

A relevant observation from our study relates to procedural workflow and hospitalization burden. Nearly all patients treated with leadless devices underwent a single-step strategy, with immediate AVN ablation followed by device implantation through the femoral approach. This streamlined workflow translated into shorter cumulative hospitalization, which is clinically meaningful in an elderly and comorbidity-laden population. Prior series have similarly reported the feasibility and safety of simultaneous AVN ablation and leadless pacemaker implantation, with low complication rates and durable pacing performance (7–9). By contrast, most patients receiving transvenous devices underwent staged procedures, prolonging hospitalization time. This difference may have health-economic implications, as shorter hospitalizations reduce costs and minimize exposure to hospital complications. While the rate of acute complications was low and not significantly different between groups, vascular access–related bleeding occurred exclusively in the leadless arm, reminding that careful management of femoral access remains critical (7). From a long-term perspective, leadless devices may also provide advantages in reducing chronic lead complications and infection risk—though their non-extractability and battery longevity remain areas requiring further evaluation, particularly in younger or lower-risk cohorts (6, 14). Taken together, our results support the role of leadless pacing as a safe and effective alternative in patients undergoing ablate-and-pace, with the choice of device best individualized based on patient profile, procedural logistics, and long-term pacing needs. In particular, leadless pacemakers offer a personalized medicine approach. Leadless pacing adds value in a defined subset of ablate-and-pace patients in whom the risk phenotype and procedural logistics favor a single-step femoral approach: elderly or frail patients, those with prior pocket/lead complications or high infection risk, difficult upper-venous access, or substantial anticoagulation/bleeding burden, in whom pocket and lead-related risks are relatively more important than the marginal difference in length of stay. Expected pacing duration and feasibility of single-step approach should always be taken into account in the precision-based selection strategy.

#### Clinical phenotyping and device selection

In the context of these findings, the key differentiator of the leadless strategy is its ability to support a streamlined single-session femoral ablate-and-pace workflow, rather than intrinsic superiority of the device itself. In our centers, this translated into a practical care pathway in which patients undergo AV node ablation and leadless pacemaker implantation during the same procedure via a single femoral venous access, preceded by systematic assessment of frailty, anticoagulation strategy, and ilio-femoral vascular suitability, and followed by standardized hemostasis, early mobilization, and short planned hospitalization. This operational gain must be balanced against a distinct risk profile, as femoral access-site bleeding events occurred only in the leadless cohort in our series, underscoring the need for meticulous access management with ultrasound-guided puncture, structured hemostasis protocols, and careful selection of patients in whom femoral access is appropriate. Long-term considerations further refine patient selection: current leadless systems offer good but finite battery longevity under near-100% pacing, are generally non-extractable, and cannot be easily upgraded to CRT or multi-chamber pacing, which favors their use in older, frail, high-comorbidity or high-infection-risk patients with limited expected device-replacement needs, while transvenous systems remain preferable in younger or lower-risk individuals in whom future extraction or system upgrade is more likely to be relevant. This study establishes a clinically derived, bedside-implementable phenotyping framework for ablate-and-pace device selection, representing a practical approach to precision cardiology in this population. The framework relies on readily available clinical variables (age, frailty, comorbidity burden, venous anatomy, infection risk, anticoagulation status, and procedural logistics) that can be systematically evaluated by any operator to guide individualized patient-device matching. However, this clinical-level precision medicine represents only the initial layer of what could become a far more granular, data-driven selection process. Future research could substantially enhance and validate this framework by integrating genomic and multi-omics markers relevant to device-related complications and anticoagulation response. Additionally, machine learning–derived risk prediction models trained on large-scale ablate-and-pace cohorts could integrate multi-dimensional clinical, imaging, device, and genomic data to generate individualized risk scores for various adverse outcomes (device infection, lead dislodgement, vascular complications, mortality) and thereby refine the choice of device type, access route, and pacing strategy for each patient.

## Limitations

The current study is a retrospective study from high-volume centers experienced in pacemaker implantation. Although the study was conducted in high-volume European centers with extensive experience in both node ablation and pacemaker implantation, the results may not be generalizable to centers with lower procedural expertise. The main limitation is lack of randomization. However, there was no independent predictor of the primary endpoint. The same experienced operators performed the procedures in both groups. Device selection was at the discretion of the implanting physician. Transvenous pacemakers can also be implanted using a single step strategy but in all the institutions included this approach is not preferred due to concerns for lead displacement/damage or loss of capture during or immediately after the ablation. The composite outcome was used for consistency across centers. AF-related hospitalizations/unplanned visits post-AVN ablation may reflect heart failure decompensation, anticoagulation issues, comorbidity, not AF *per se*. Finally, the present study operates at the level of clinical-phenotype–based precision medicine and does not incorporate genomic, multi-omics, or artificial intelligence–driven risk stratification. We did not evaluate, for example, genetic variants that may predispose to device infection or vascular complications, pharmacogenomic determinants of anticoagulation safety, or machine learning–derived scores for individualized device selection and procedural strategy. Future work could validate and augment our phenotyping framework by integrating such genomic and AI-based tools, potentially improving prediction of leadless-related complications (e.g., femoral access bleeding), lead-related complications (e.g., infection, displacement), optimizing anticoagulation management in the perioperative period, and refining patient-specific recommendations for ablate-and-pace workflows.

## Conclusions

In patients with symptomatic permanent AF undergoing ablate-and-pace, both leadless and conventional single-chamber transvenous pacemakers demonstrated similar safety and efficacy outcomes. Effectiveness was inferred primarily from event-free survival. Leadless system allowed for a streamlined single-step approach with reduced hospitalization time, representing a clinically meaningful advantage in an elderly, comorbidity-burdened population.

## Data Availability

The raw data supporting the conclusions of this article will be made available by the authors, without undue reservation.

## References

[1] Hindricks G Potpara T Dagres N Arbelo E Bax JJ Blomström-Lundqvist C 2020 ESC guidelines for the diagnosis and management of atrial fibrillation developed in collaboration with the European association for cardio-thoracic surgery (EACTS). Eur Heart J. (2021) 42:373–498. Oxford University Press. 10.1093/eurheartj/ehaa612 32860505

[2] Kirchhof P Camm AJ Goette A Brandes A Eckardt L Elvan A Early rhythm-control therapy in patients with atrial fibrillation. N Engl J Med. (2020) 383:1305–16. 10.1056/NEJMoa2019422 32865375

[3] Wyse DG Waldo AL DiMarco JP Domanski MJ Rosenberg Y Schron EB A comparison of rate control and rhythm control in patients with atrial fibrillation. N Engl J Med. (2002) 347:347. 10.1056/NEJMoa021328 12466506

[4] Vlachos K Letsas KP Korantzopoulos P Liu T Efremidis M. Sideris A: a review on atrioventricular junction ablation and pacing for heart rate control of atrial fibrillation. J Geriatr Cardiol. (2015) 12:547–54. 10.11909/j.issn.1671-5411.2015.05.005 26512247 PMC4605951

[5] Brignole M Menozzi C Gianfranchi L Musso G Mureddu R Bottoni N Assessment of atrioventricular junction ablation and VVIR pacemaker versus pharmacological treatment in patients with heart failure and chronic atrial fibrillation. Circulation. (1998) 98:953–60. 10.1161/01.CIR.98.10.953 9737514

[6] Reddy VY Knops RE Sperzel J Miller MA Petru J Simon J Permanent leadless cardiac pacing. Circulation. (2014) 129:1466–71. 10.1161/CIRCULATIONAHA.113.006987 24664277

[7] Okabe T El-Chami MF Lloyd MS Buck B Gornick CC Moore JC Leadless pacemaker implantation and concurrent atrioventricular junction ablation in patients with atrial fibrillation. Pacing Clin Electrophysiol. (2018) 41:504–10. 10.1111/pace.13312 29476660

[8] El-Chami MF Shinn T Bansal S Martinez-Sande JL Clementy N Augostini R Leadless pacemaker implant with concomitant atrioventricular node ablation: experience with the Micra transcatheter pacemaker. J Cardiovasc Electrophysiol. (2021) 32:832–41. 10.1111/jce.14881 33428248 PMC7986103

[9] Martínez-Sande JL Rodríguez-Mañero M García-Seara J Lago R González-Melchor L Kreidieh B Acute and long-term outcomes of simultaneous atrioventricular node ablation and leadless pacemaker implantation. Pacing Clin Electrophysiol. (2018) 41:1484–90. 10.1111/pace.13496 30221378

[10] Boveda S Higuera L Longacre C Wolff C Wherry K Stromberg K Two-year outcomes of leadless vs. Transvenous single-chamber ventricular pacemaker in high-risk subgroups. Europace. (2023) 25:1041–50. 10.1093/europace/euad016 36757859 PMC10062361

[11] Glikson M Nielsen JC Kronborg MB Michowitz Y Auricchio A Barbash IM ESC Guidelines on cardiac pacing and cardiac resynchronization therapy. Eur Heart J. (2021) 2021:1–94. 10.1093/eurheartj/ehab364 34586378

[12] Chatterjee NA Upadhyay GA Ellenbogen KA McAlister FA Choudhry NK Singh JP. Atrioventricular nodal ablation in atrial fibrillation a meta-analysis and systematic review. Circ Arrhythm Electrophysiol. (2012) 5:68–76. 10.1161/CIRCEP.111.967810 22187425

[13] Kirkfeldt RE Johansen JB Nohr EA Jorgensen OD Nielsen JC. Complications after cardiac implantable electronic device implantations: an analysis of a complete, nationwide cohort in Denmark. Eur Heart J. (2014) 35:1186–94. 10.1093/eurheartj/eht511 24347317 PMC4012708

[14] Reynolds D Duray GZ Omar R Soejima K Neuzil P Zhang S A leadless intracardiac transcatheter pacing system. N Engl J Med. (2016) 374:533–41. 10.1056/NEJMoa1511643 26551877

